# Exam anxiety and associated factors among Palestinian university students

**DOI:** 10.3389/fpsyg.2026.1776930

**Published:** 2026-05-26

**Authors:** Issa Abuiram, Noor Hassline Mohamed, Praneetha Palasuberniam

**Affiliations:** Department of Public Health, Universiti Malaysia Sabah, Kota Kinabalu, Malaysia

**Keywords:** exam anxiety, Palestine, social determinants, university students, well-being

## Abstract

**Background:**

Exam anxiety is a specific form of performance anxiety in which students experience intense worry, physical arousal, and unhelpful behavior around exam situations. Exam anxiety is common in higher education and is associated with poor academic performance. Additional political and economic strain may increase anxiety and threaten achievement at Palestinian universities.

**Aim:**

This study aimed to investigates exam anxiety and associated factors, such as lifestyle and socio-demographic factors, among Palestinian university students.

**Methods:**

This cross-sectional descriptive study was conducted among students from three institutions in Palestine (Al-Quds University, Hebron University, and An-Najah National University), with 1,519 participants completing the survey. Data were collected using self-administered questionnaire; Westside Test Anxiety Scale (WTAS) was used to assess exam anxiety. Descriptive statistics and general linear model were performed to identify factors associated with exam anxiety.

**Results:**

Among the 1,519 participants, a significant majority (61.8%) reported elevated exam anxiety levels, with nearly one-third (32.5%) categorized as experiencing “extremely high” anxiety. In contrast, only 38.2% of respondents fell within the lower end of the anxiety spectrum (from low to high normal). Student anxiety arises from a complex interplay of sociodemographic, academic, and lifestyle factors, with the overall model accounting for 21.2% of the variance. The most significant predictors of high anxiety include intense academic overload, lower family income (below $1,140), smoking, and physical illness. Conversely, strong family support and high academic performance (GPA of 80 or above) serve as essential protective factors. Additionally, lifestyle choices are influential, as students who exercise only 1 to 2 days per week report higher anxiety levels compared to those who engage in more frequent exercise. Furthermore, institutional differences are evident, with students from Al-Quds University exhibiting higher exam anxiety levels than those from An-Najah University.

**Conclusion:**

The high prevalence of exam anxiety among Palestinian university students highlights the need for systemic institutional support. Universities should implement regular mental health screenings and offer specialized counseling, especially for those in high-stress academic fields. Additionally, reforming assessment methods to reduce academic overload and promoting consistent physical activity are essential evidence-based strategies for alleviating distress. These measures can contribute to a more balanced academic environment and enhance students’ overall well-being.

## 1 Introduction

Universities play a critical role in shaping students’ long-term health outcomes by fostering their social, emotional, and mental well-being alongside providing education (Skoglund et al., 2021; Wizemann, 2021). Robust health policies and support systems within universities are fundamental for student well-being and serve as a cornerstone of community public health advances (Gopalan, 2023; Cage, 2021; Khatri et al., 2024). Conversely, students with better mental health tend to show higher academic motivation and more sustained engagement in their studies (Mahdavi et al., 2023).

Higher education is consistently linked to lower rates of chronic diseases, healthier behaviors, and a reduced risk of mortality (Balaj et al., 2024; Zouine et al., 2024; Scroggs et al., 2025). Simultaneously, many students desire to succeed and take advantage of all available educational opportunities when they enroll in college, yet a significant percentage struggle to achieve these goals (Kauser et al., 2021). These difficulties are often driven by factors such as anxiety, elevated stress, and ineffective time management (McCurdy et al., 2022; Rudland et al., 2020; Yu, 2023; Fu et al., 2025).

Exam anxiety is a specific form of anxiety that arises in evaluative contexts, particularly during examinations (Putwain and Symes, 2020; Sommer and Arendasy, 2014). This condition is strongly associated with poorer academic outcomes, including lower standardized test scores, weaker university entrance results, and reduced grade point averages (von der Embse et al., 2018). A significant amount of research shows that heightened test anxiety is associated with reduced academic performance (Benjamin and Mohammed, 2023; Hyseni Duraku and Hoxha, 2018). In particular, it has been shown that academic anxiety raises avoidance, self-doubt, and fear of failure; these consequences weaken students’ resilience and restrict their involvement in their studies, leading to passive procrastination (Hsu and Goldsmith, 2021). Furthermore, exam anxiety is recognized as a significant and widespread threat to student well-being in higher education (Tan et al., 2023).

Grounded in the control-value theory, this framework suggests that two main factors drive academic emotions: subjective control and subjective value. Exam anxiety arises when a student places a high value on success but perceives low control over the outcome (Pekrun, 2006). In a university context, external stressors often intensify this anxiety. As degrees become more expensive and the job market becomes more competitive, the value of passing an exam is artificially inflated (Pekrun and Stephens, 2010). This high-pressure environment diminishes a student’s sense of control, leading to increased exam anxiety. However, Attentional Control Theory suggests that exam anxiety primarily impairs processing efficiency rather than just performance quality (Eysenck et al., 2007). The text suggests that thoughts driven by anxiety and worry deplete working memory resources and disrupt essential executive functions such as inhibition and shifting (Corbetta and Shulman, 2002). To compensate for their anxiety, students often need to invest significantly more cognitive effort to keep their grades, leading to a greater mental cost for achieving the same academic results (Basten et al., 2011).

Rates of exam anxiety vary among nations. For example, about 18% in Malaysia (Alamri and Nazir, 2022) and approximately 50 to 70% in Ethiopia (Tsegay et al., 2019; Kassaw et al., 2024). In addition, about one-third of students in China, Saudi Arabia, and the United Arab Emirates reported unhealthy levels of exam anxiety (Liu et al., 2021; Memon et al., 2023; Jirjees et al., 2024).

Palestinian university students face routine academic demands in chronic sociopolitical and economic adversity (Mahamid and Bdeir, 2022; Sabbah et al., 2025). Recent evidence indicates a high prevalence of psychological distress among Palestinian university students, with anxiety and depression rates reaching 60% due to exposure to political violence and insecurity (Ahmead et al., 2024; Khalili et al., 2025). The contextual dimension of student well-being extends beyond individual psychological factors and is significantly shaped by the macro-level conditions of the Palestinian territories. Armed conflicts and violence have a detrimental impact on local universities, resulting in institutional shutdowns, the repurposing of facilities for military use, and loss of life within the academic community (Assefa et al., 2022).

In the West Bank, a system of checkpoints and restricted mobility worsens the situation, leading to frequent absences among teachers and students and resulting in a fragmented educational experience (Jones and Naylor, 2014). The systemic instability experienced by students fosters a state of hypervigilance, resulting in chronic stress from unpredictable access to campus. This situation is associated with increased symptoms of anxiety, depression, and somatic issues (Timmerman and Volpe, 2023). Furthermore, the existing literature on exam anxiety primarily focuses on politically stable regions, which creates a significant knowledge gap regarding high-conflict areas like Palestine. There is a considerable lack of understanding about the unique psychological pressures that students experience in such prolonged conflict settings. To address this gap, the current study investigates exam anxiety and associated factors, such as lifestyle and socio-demographic factors, among Palestinian university students.

## 2 Materials and methods

### 2.1 Study design

This study used an institutional-based cross-sectional design.

### 2.2 Study context and period

Three major Palestinian universities, Hebron University, Al-Quds University, and An-Najah National University, were selected as the study setting due to their large student populations and their representation of the southern, central, and northern regions of the West Bank, respectively. Collectively, these universities offer a diverse multidisciplinary environment, encompassing over 35 faculties and a wide array of undergraduate and postgraduate programs. These include specialized degrees in medicine, life sciences, engineering, and the humanities, ensuring a representative sample of the Palestinian higher education landscape (Ahmead et al., 2024). This research conducted from August 25 to November 14, 2025, utilizing a sample that reflects the demographic profiles of the students.

### 2.3 Participant recruitment

A convenience sampling strategy, a non-probability sampling method selected for its practical suitability in the present research context (Stratton, 2021). Although probability sampling is ideal, it was infeasible due to enduring sociopolitical instability within the Palestinian territories. In the West Bank, access is often hindered by macro-level constraints such as restricted mobility, checkpoints, and unpredictable institutional shutdowns (Mahamid and Bdeir, 2022). This method enabled data collection during a narrow window of accessibility across a population of 133,500 students [Palestinian Central Bureau of Statistics (PCBS), 2023]. To ensure statistical power, we used Cochran’s formula to calculate the sample size. With 95% confidence level (Z = 1.96) and a rigorous margin of error of 2.5% (e = 0.025), assuming an expected proportion (P) of 0.5 to ensure maximum sample variability. Given the total population (N) of 133,500 across the institutions, the final sample size is 1,519 students (Daniel and Cross, 2018). Attrition was effectively managed at the point of entry through the informed consent protocol, which required the submission of all survey items. The final dataset is complete, with no missing values, and represents a complete-case sample.

$$\text{Sample size}=\frac{\left(\frac{\left(Z^{2}P\;(1-P)\right)}{e^{2}}\right)}{\begin{array}{c} \frac{1+(Z2P\;(1-P)}{e2N} \end{array}}=1,519\;\text{participants}$$

### 2.4 Study variable

*Dependent variable:* Exam anxiety.

*Independent variable:* Sociodemographic characteristics include institutional affiliation, field of study, gender, age, study year, place of living and area. Economic indicators such as family monthly salary and financial support. Academic factors for example, Grade Point Average (GPA) and perceived academic overload. Furthermore, health and lifestyle behaviors, including physical morbidity, tobacco use, and physical activity status. Finally, family support as a social determinant.

### 2.5 Study tools

#### 2.5.1 Socio-demographic and related determinants of the participants

To identify potential correlates of the primary outcome, this study examined participants’ socio-demographic backgrounds. The factors include institutional affiliation, field of study, gender, age, study year, place of living and area. Economic factors were captured via parents’ monthly income and financial support, while academic standing was measured through Grade Point Average (GPA) and overload. According to the GPA Calculator (2025), students’ cumulative averages in Palestinian universities are often classified as excellent (90–100%), very good (80–89%), good (70–79%), and fair (60–69%). Physical morbidity and tobacco use (classified as yes or no) were examples of health and lifestyle characteristics (Jarso et al., 2024). Moreover, physical activity was assessed using a descriptive rating scale in which participants reported the number of days they engaged in at least 30 min of moderate-intensity physical activity (Milton et al., 2013). Social influence was measured based on the presence or absence of family support (Başaslani, 2022).

#### 2.5.2 The Westside test anxiety scale (WTAS)

This 10-item instrument is designed to evaluate the severity of test anxiety among adolescents and adults. The scale encompasses two main components: six items measuring impairment and cognitive interference (the extent to which anxiety disrupts thinking and performance) and four items assessing self-anxiety (the subjective experience of nervousness). The total score is divided by ten to produce an average score ranging from 1.0 to 5.0, corresponding to six established severity levels from comfortably low-test anxiety (1.0–1.9) to extremely high anxiety (4.0–5.0) as outlined by Driscoll (2007). The WTAS has demonstrated strong psychometric properties, with reported internal consistency coefficients of Cronbach’s alpha = 0.87 and split-half reliability up to 0.94 across diverse international samples (Tsegay et al., 2019; Talwar et al., 2019; Kassaw et al., 2024).

### 2.6 Procedure

Due to travel restrictions in the West Bank, data were collected online via an anonymous Google Forms questionnaire. The survey link was shared through university emails, Facebook groups, and WhatsApp networks across three universities. Each link included an invitation explaining the study’s purpose. Before starting the survey, all participants provided electronic informed consent; the system only allowed those who consented to proceed. A total of 1,519 students voluntarily completed the questionnaire. All responses remained anonymous, and participants were informed they could withdraw at any time.

### 2.7 Data analysis

Statistical analyses were performed using IBM SPSS Statistics (version 25.0; IBM Corp., Armonk, NY, United States). Data normality was assessed using the Kolmogorov–Smirnov test and visual inspection of histograms. Outliers were identified and visualized using boxplots. Descriptive statistics, including frequencies, percentages, means, and standard deviations, were used to summarize participant sociodemographic characteristics and study variables. To evaluate the internal consistency of the measurement scale, Cronbach’s alpha (*α*) was calculated. A multiple linear regression analysis was conducted to examine the relationships between variables. For all analyses, a *p*-value of < 0.05 was considered statistically significant.

### 2.8 Ethical considerations

The study protocol was reviewed and approved by the Medical Research Ethics Committee of the Faculty of Medicine and Health Sciences, University Malaysia Sabah (reference no: UMS FPSK 6.9/100-6/1/95). Administrative approval was also obtained from the participating universities prior to data collection. Additionally, participants were briefed on the study’s objectives and provided written informed consent. The consent process emphasized that participation was voluntary, and respondents were assured of data confidentiality and their right to withdraw from the study at any time without penalty.

## 3 Results

### 3.1 Socio-demographic characteristics of the participants

The demographic characteristics of the 1,519 study participants highlight a diverse group heavily skewed towards female students. Three universities provided participants: Hebron University (28.6%), Al-Quds University (32.8%), and An-Najah National University (38.6%). 21.1% of students are majoring in medical and health, followed by the sciences (34.9%), humanities and law (31.3%), and technical and applied (12.8%). There was a notable gender imbalance, with females constituting a large majority (69.7%) compared to males (30.3%). The largest age group was 20–21 years (40.9%), and students were almost evenly split between the first or second year (43.2%) and the third or fourth year (43.1%). In terms of socio-economic status, the majority reported a moderate family monthly salary (41.6%), and a significant majority (77.0%) were unfunded regarding financial support. The participants were nearly equally split between living in a city (47.2%) and a village (46.5%), with the Southern area contributing the most participants (39.8%), as shown in Table 1.

**Table 1:** Socio-demographic and economic characteristics of participants (*n* = 1,519).

Participants characteristics	(*n*)	%
Institutional affiliation		
Hebron University	434	28.6%
Al-Quds University	499	32.8%
An-Najah University	586	38.6%
Field of study		
Medicine and health	320	21.1%
Sciences	530	34.9%
Humanities and law	475	31.2%
Technical and applied	194	12.8%
Gender		
Male	460	30.3%
Female	1,059	69.7%
Age of participants		
17–19 year	569	37.5%
20–21 year	622	40.9%
22 and older	328	21.6%
Study year		
First or second year	656	43.2%
Third or fourth year	655	43.1%
Fifth and higher year	208	13.7%
Place of living		
City	718	47.2%
Village	706	46.5%
Refugee camp	95	6.3%
Area		
Southern area	604	39.8%
Middle area	360	23.7%
Northern area	555	36.5%
Family monthly salary (US $)		
Low (< 570)	490	32.3%
Moderate (570–1,140)	632	41.6%
High (> 1,140)	397	26.1%
Financial support		
Funded	350	23.0%
Unfunded	1,169	77.0%

### 3.2 Academic, health and lifestyle characteristics of the participants

The academic, health and lifestyle profiles of the participants (*n* = 1,519) are summarized in Table 2. Regarding academic performance, the largest segment of participants reported GPA between 80 and 89% (45.6%), while only 8.5% reported as poor achievement. When assessing workload demands, the most common response was “mostly true” (44.4%). Regarding health and lifestyle factors, 10% of participants reported having a physical morbidity. Concerning tobacco use, 21.7% identified as smokers, while 78.3% indicated they did not smoke. Almost half of the respondents (48.0%) reported engaging in at least 30 min of vigorous physical activity on 3–4 days per week, with 24.6% doing so on 5 or more days, whereas only 5.1% indicated no physical activity at all. Finally, a large majority (77.9%) reported receiving family support.

**Table 2:** Academic and health-related characteristics (*N* = 1,519).

Academic and health-related characteristics	Frequency	Percentage
Grade point average (GPA)		
90 and higher/Excellent	344	22.7%
80–89/Very good	693	45.6%
70–79/Good	353	23.2%
60–69/Fair	129	8.5%
My workload is so demanded		
Not at all true	532	35%
Mostly true	674	44.4%
Very true	313	20.6%
Physical morbidity		
Present	152	10%
Absent	1,367	90.0%
Tobacco use status		
Smoker	329	21.7%
Non-Smoker	1,190	78.3%
Days with 30 + min vigorous physical activity		
0 day	78	5.1%
1–2 days	338	22.3%
3–4 days	729	48.0%
5 + days	374	24.6%
Family support availability		
Present	1,183	77.9%
Absent	336	22.1%

### 3.3 The psychometric properties for (WTAS)

The Westside Test Anxiety Scale (WTAS) total score, consisting of 10 items, demonstrated excellent reliability with a Cronbach’s *α* of 0.90, a mean of 34.23, and a standard deviation of 9.46. Regarding the subscales, the Worry subscale (6 items) showed high internal consistency (α = 0.85, M = 20.05, SD = 5.95), while the Impairment subscale (4 items) also reached an acceptable reliability level (α = 0.79, M = 14.18, SD = 3.97), as seen in Table 3.

**Table 3:** Psychometric properties for scales (*N* = 1,519).

Variables	Cronbach’s α	M	SD	Variance	Items (*N*)
WTAS total	0.90	34.23	9.46	89	10
Worry subscale	0.85	20.05	5.95	35	6
Impairment subscale	0.79	14.18	3.97	15.7	4

M = Mean, SD = Standard Deviation, WTAS = Westside Test Anxiety Scale.

### 3.4 Prevalence and severity categorization of exam anxiety

**Figure 1:**
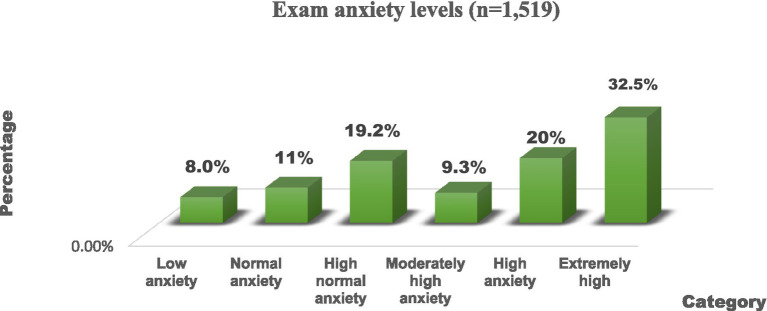
Breakdown of exam anxiety scores (*N* = 1,519).

The updated data, derived from Figure 1, indicate a clear positive skew toward higher levels of anxiety among a sample of 1,519 participants. A significant majority of participants, 61.8%, reported experiencing anxiety levels classified as moderately high to extremely high. The highest percentage of respondents, at 32.5% (*n* = 494), fell into the “Extremely high” anxiety category. Twenty percent (*n* = 303) of participants reported experiencing high anxiety, while 9.3% (*n* = 141) were categorized as having moderately high anxiety. In contrast, more than a quarter of the individuals (38.2%) had all three of the lowest anxiety levels (low, normal, and high normal), at 8% (*n* = 121), 11% (*n* = 167), and 19.2% (*n* = 293), respectively.

### 3.5 Comparative analysis of exam anxiety across demographic groups

The analysis of demographic variables revealed that field of study is the most significant determinant of exam anxiety (*F* = 48, *p* < 0.001), with students in medicine and health reported the highest mean score (M = 38.53), followed by those in the Sciences (M = 35.19) and humanities and law (M = 31.81) and the last the technical and applied fields (M = 30.48). Significant differences were also observed based on institutional affiliation (*F* = 7.96, *p* = 0.001), where students at Al-Quds University exhibited higher anxiety levels (M = 34.97) than those at Hebron University (M = 32.72). Furthermore, family monthly salary played a critical role (*F* = 4.69, *p* = 0.009); students from low-income reported significantly higher anxiety (M = 35.13) than their high-income counterparts (M = 33.15). Regarding the non-significant factors, the analysis indicates that gender, age, study year, place of living, area and financial support had no significant effect, as seen in Table 4 and Supplementary Table S2.

**Table 4:** Comparative analysis of exam anxiety across socio-demographic categories (*N* = 1,519).

Demographic variables	Category	Exam anxiety		
		Mean ± SD	T/F	*p*-value
Institutional affiliation	Hebron University	32.72 ± 9.26	7.96	**0.001**
	Al-Quds University	34.97 ± 9.26		
	An-Najah University	34.74 ± 9.66		
Field of study	Medicine and health	38.53 ± 8.52	48.56	**<0.001**
	Sciences	35.19 ± 8.65		
	Humanities and law	31.81 ± 9.14		
	Technical and applied	30.48 ± 10.6		
Gender	Male	34.51 ± 9.56	0.763	0.445
	Female	34.11 ± 9.42		
Age	18–19 year	33.97 ± 9.40	0.819	0.414
	20–21 year	34.18 ± 9.52		
	22 and older	34.80 ± 9.45		
Study year	First or second year	33.76 ± 9.57	1.67	0.187
	Third or fourth year	34.47 ± 9.27		
	Fifth and higher year	34.99 ± 9.70		
Place of living	City	34.43 ± 9.46	1.96	0.141
	Village	33.84 ± 9.43		
	Camp	35.73 ± 9.60		
Area	Southern area	33.72 ± 9.41	1.92	0.147
	Middle area	34.93 ± 9.49		
	Northern area	34.35 ± 9.47		
Family monthly salary	Low (< 570)	35.13 ± 9.28	4.69	**0.009**
	Moderate (570–1,140)	34.22 ± 9.15		
	High (> 1,140)	33.15 ± 10.0		
Financial support	Funded	34.13 ± 9.31	0.783	0.434
	Unfunded	34.58 ± 9.95		

The independent t-test (T) compares means between two groups, while analysis of variance (F) compares three or more. Standard deviation (DS) measures how much individual data points vary from those means. Values in bold indicate statistical significance at the *p* < 0.05 level.

### 3.6 Comparative analysis of exam anxiety across related factors (*N* = 1,519)

The analysis of academic, health and life style variables demonstrates that several key factors are highly significant predictors of exam anxiety (*p* ≤ 0.001). A strong inverse relationship was observed between grade point average and anxiety levels (*F* = 22.2, *p* < 0.001); students with lower academic performance (60–69) reported the highest anxiety (M = 38.82), while those with high achievement (80–89) reported significantly lower levels (M = 32.86). This finding is closely mirrored by the impact of academic workload (*F* = 116, *p* < 0.001), where students who perceived their workload as “very true” to be demanding experienced peak anxiety (M = 39.27), compared to those who felt it was “not at all true” (M = 30.0).

Social, health and life style factors also emerged as critical determinants. Students with physical morbidity had higher mean (M = 37.88). Furthermore, those who identified as smokers (M = 36.96) exhibited significantly greater anxiety than non-smoking counterparts (*p* = 0.001). Physical activity showed a protective effect (*F* = 13.3, *p* = 0.001); students who engaged in vigorous activity for five or more days per week reported the lowest anxiety (M = 32.6), whereas those with only 1–2 days of activity reported higher mean (M = 36.82). Finally, the absence of family support was associated with significantly higher anxiety level (M = 36.85) than students who did not have support from their families (M = 33.45, *p* = < 0.001) (see Table 5).

**Table 5:** Comparative analysis of exam Anxiety across related factors (*N* = 1,519).

Variables	Categories	Exam anxiety		
		Mean ± SD	T/F	*p*-value
Grade point average	≥ 90/Excellent	33.27 ± 10.2	22.2	**< 0.001**
	80–89/Very good	32.86 ± 9.35		
	70–79/Good	36.21 ± 8.23		
	60–69/Fair	38.82 ± 8.95		
My workload is so demanded	Not at all true	30.00 ± 9.54	116.6	**< 0.001**
	Mostly true	35.25 ± 8.32		
	very true	39.27 ± 8.57		
Physical morbidity	Present	37.88 ± 8.88	5.07	**0.001**
	Absent	33.82 ± 9.44		
Tobacco use status	Smoker	36.96 ± 8.99	6.14	**< 0.001**
	Non-smoker	33.48 ± 9.45		
Days with 30 + min vigorous physical activity	0 day	35.60 ± 10.61	13.1	**0.001**
	1–2 days	36.82 ± 8.35		
	3–4 days	33.73 ± 9.23		
	5 + days	32.60 ± 10.1		
Family support	Present	33.45 ± 9.56	6.14	**<0.001**
	Absent	36.85 ± 8.62		

Values in bold indicate statistical significance at the *p* < 0.05 level.

### 3.7 Determinants of exam anxiety: a multiple linear regression analysis

A multiple linear regression analysis was conducted to identify the predictors of exam anxiety among Palestinian university students. Before the analysis, the assumptions of the General Linear Model were verified. The independence of the residuals was confirmed with a Durbin-Watson statistic of 2.016 (Field, 2024). To assess multicollinearity among the independent variables, Variance Inflation Factors (VIF) and tolerance statistics were utilized. The results indicated that the VIF values for all predictors ranged from 1.027 to 1.134, which is significantly lower than the conservative threshold of 5.0 (Alin, 2010).

The results indicated that the model was statistically significant and provided a robust fit to the data, *F* (9, 1,508) = 44.897, *p* < 0.001 (Supplementary Table S5). The general model explained 21.2% of the total variance (R^2^ = 0.212) and had an intercept of B = 35.88, as shown in Supplementary Tables S3, S4. The effect sizes for each predictor are reported as Partial ETA Squared, with academic overload showing the strongest impact on exam anxiety (n^2^_p_ = 0.069, *p* < 0.001; Cohen, 2013).

To assess the influence of outliers on the model, Cook’s Distance and Centered Leverage values were calculated. The maximum Cook’s distance was 0.017, which is well below the threshold of 1.0 (Cook and Weisberg, 1982). The maximum centered leverage value was 0.019, falling within the acceptable limits for a large sample size (*N* = 1,519). These diagnostics confirm that the regression coefficients are stable and were not disproportionately influenced by any single observation (Stevens, 2002). As shown in Supplementary Tables S3, S6.

**Figure 2:**
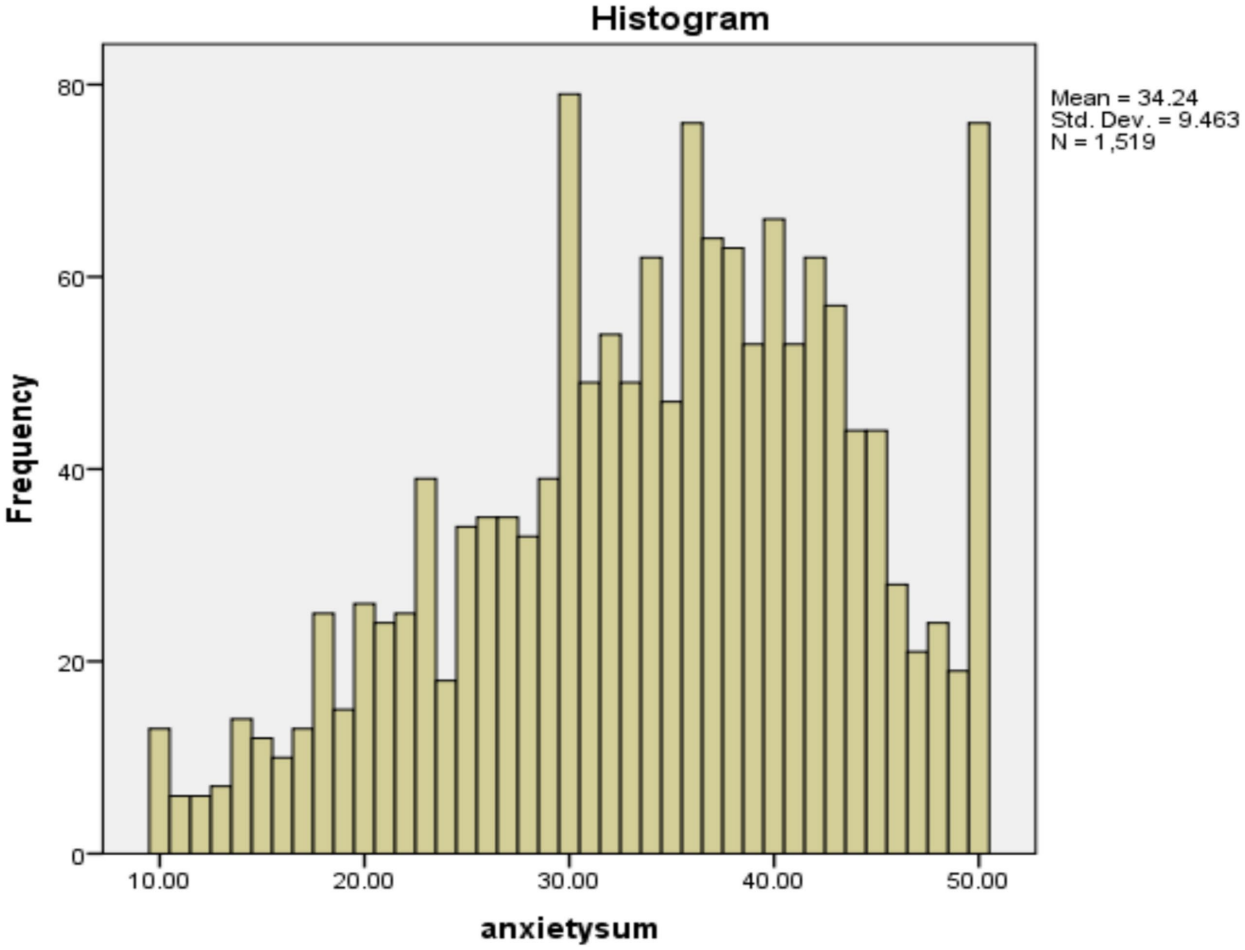
Histogram of the total exam anxiety scores (*N* = 1,519).

**Figure 3:**
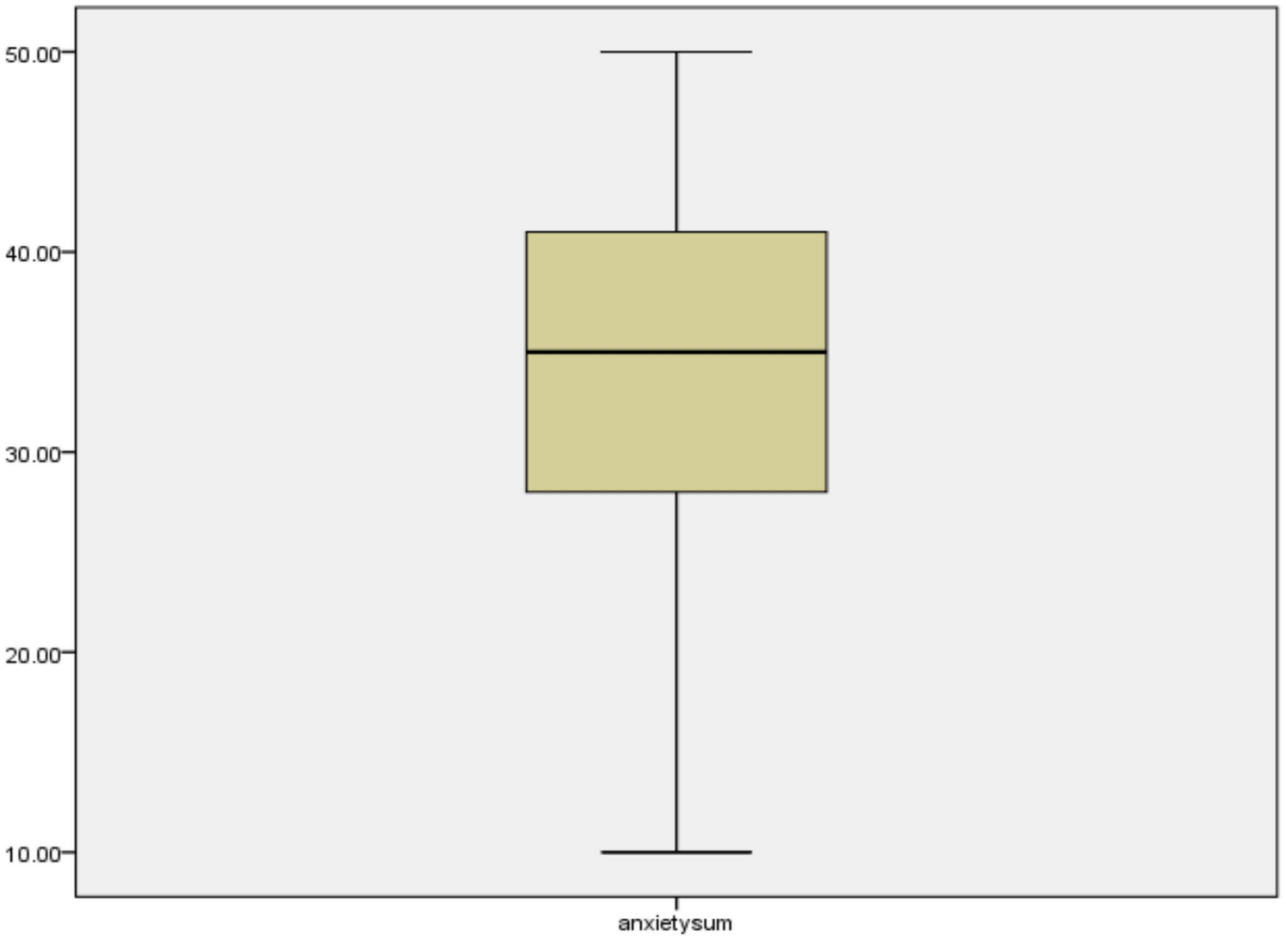
Boxplot of exam anxiety scores (*N* = 1,519).

The histogram illustrates the distribution of total exam anxiety scores among the study participants. The data exhibit a unimodal, approximately normal distribution, supported by a mean of 34.23 and a median of 35.00, as shown in Figure 2 and Supplementary Table S5. Given the large sample size, the distribution’s shape indicates that the scores cluster around the mean with a slight negative skew. A boxplot was utilized to screen the data for univariate outliers prior to conducting the regression analysis. The median score (35.00) is centered within the box, indicating a symmetric distribution of anxiety levels. Crucially, no data points extend beyond the upper or lower whiskers (1.5 times the interquartile range), confirming the total absence of outliers as shown in Figure 3.

The findings suggest that students enrolled at Al-Quds University exhibited higher anxiety levels (B = 1.4) than their counterparts at An-Najah University. Moreover, students from low-income (B = 2.02) and moderate-income (B = 1.82) households experienced significantly greater anxiety compared to those from high-income backgrounds.

Students in medicine and health (B = 4.56) and science programs (B = 3.03) demonstrated the highest anxiety levels, significantly exceeding those of their peers in technical fields. Conversely, academic achievement appeared to serve as a buffer against distress; a negative correlation was observed between GPA and anxiety. Specifically, students with ‘Excellent’ (B = −3.02) and “Very satisfactory” (B = −3.22) reported lower anxiety levels compared to those in the ‘Fair’ (60–69%) category. Physical morbidity (B = 1.65) and tobacco use (B = 1.77) are significant predictors of increased anxiety. In contrast, family support acts as a crucial buffer, significantly lowering anxiety scores (B = −1.81). The relationship between physical activity and anxiety levels is complex. Specifically, engaging in moderate activity for 1–2 days per week is associated with higher anxiety (B = 2.45) compared to the most active students.

The perceived workload emerged as the most significant predictor in the model. Students who indicated a low workload burden, marked as “Not at all,” experienced considerably lower anxiety levels (B = −6.80) compared to those who described their burden as “Very True” (refer to Table 6 and Supplementary Table S2).

**Table 6:** Multiple linear regression model of factors associated with exam anxiety.

Parameters	B	Std. error	t	Sig	95% Cl		Tolerance	VIF	Partial eta squared
Intercept	35.88	1.226	29.25	< 0.001	33.47	38.28			0.363
Institutional affiliation									
Hebron University	0.172	0.566	0.304	0.761	−0.761	1.282	0.920	1.087	0.000
Al-Quds University	1.400	0.557	2.514	0.012	0.307	2.492			0.004
An-Najah University	Reference								
Family monthly salary									
Low (< 570)	2.022	0.584	3.460	0.001	0.876	3.168	0.974	1.027	0.008
Moderate (570–1,140)	1.825	0.546	3.341	0.001	0.754	2.897			0.007
High (> 1,140)	Reference								
Study field									
Medicine and health	4.566	0.798	5.723	<0.001	3.001	6.131	0.884	1.132	0.021
Sciences	3.039	0.731	4.157	<0.001	1.605	4.473			0.011
Humanities and law	1.062	0.725	1.466	0.143	−0.359	2.484			0.001
Technical and applied	Reference								
GPA									
≥ 90 (Excellent)	−3.026	0.914	−3.311	< 0.001	−4.818	−1.233	0.893	1.120	0.007
80–89 (Very good)	−3.224	0.874	−3.689	<0.001	−4.938	−1.509			0.009
70–79 (Good)	−0.944	0.887	−1.064	0.287	−2.685	0.796			0.001
60–69 (Fair)	Reference								
Physical morbidity									
Present	1.657	0.758	2.185	0.029	0.170	3.144	0.924	1.083	0.003
Absent	Reference								
Tobacco use status									
Smoker	1.773	0.556	3.191	< 0.001	0.683	2.863	0.918	1.089	0.007
Non-Smoker	Reference								
Family support availability									
Present	−1.814	0.535	−3.389	0.001	−2.863	−0.764	0.964	1.038	0.008
Absent	Reference								
Days with 30 + min vigorous physical activity									
0 day	0.121	1.072	0.113	0.910	−1.982	2.223	0.936	1.069	0.000
1–2 days	2.458	0.649	3.786	< 0.001	1.184	3.731			0.009
3–4 days	0.532	0.551	0.965	0.335	−0.549	1.613			0.011
5 + days	Reference								
My workload is so demanded									
Not at all true	−6.805	0.647	−10.520	< 0.001	−8.075	−5.536	0.882	1.134	0.069
Mostly true	−2.493	0.616	−4.049	< 0.001	−3.700	−1.285			0.011
Very true	Reference								

## 4 Discussion

This study aimed to investigate exam anxiety and associated factors, such as lifestyle and socio-demographic factors, among Palestinian university students.

### 4.1 Exam anxiety levels

The study found that a significant majority of respondents 61.8% of students experienced moderately high to extremely high anxiety. This finding is similar to the results of previous studies, which reported a high prevalence rate among university students in Saudi Arabia 65% (Khoshhal et al., 2017), Ethiopia [68.9% (Kassaw et al., 2024), and in the UAE 64.6% (Jirjees et al., 2024)]. However, this result is notably higher than the anxiety prevalence reported in other regional studies, such as 33.6% in another Saudi Arabia study (Almutairi et al., 2024), 55.2% in Egypt (Atef Mahmoud Mohmed et al., 2025), and 52.30% in Addis Ababa (Tsegay et al., 2019). Conversely, the current finding is lower than the exceptionally high rate of 83% reported in an Indian study (Manisha et al., 2019).

Control-Value Theory (Pekrun, 2006) explains these levels through the imbalance between high academic value and low perceived control. In Palestine, this is exacerbated by chronic sociopolitical adversity (Mahamid and Bdeir, 2022). While the stakes of success are inflated by a competitive job market (Pekrun and Stephens, 2010), student agency is undermined by systemic instability, restricted mobility, and a fragmented educational experience (Jones and Naylor, 2014). This environment fosters hypervigilance and chronic stress (Timmerman and Volpe, 2023). Moreover, Attentional Control Theory (Eysenck et al., 2007) asserts that anxiety, prevalent in areas impacted by political violence (Ahmead et al., 2024), diminishes processing efficiency by exhausting working memory resources and disrupting executive functions (Corbetta and Shulman, 2002). Therefore, these students experience an increased mental burden, requiring extraordinary cognitive exertion to cover the resources depleted by academic stress and environmental trauma (Basten et al., 2011).

### 4.2 Sociodemographic determinants of exam anxiety among participants

The institutional and academic pressure is highlighted by significant differences among universities. Students at Al-Quds University reported higher anxiety levels than those at Hebron or An-Najah universities. The results of the multivariable analysis confirm that academic discipline is a critical predictor of exam anxiety. The significantly higher scores among medical and health students (*p* < 0.001) align with previous literature (Memon et al., 2023), suggesting that the rigorous nature of medical education and the high stakes associated with clinical competence create a uniquely stressful environment.

The significant influence of socioeconomic status on exam anxiety underscores the role of financial security in academic well-being. As Jirjees et al. (2024) observed, students from lower-income households often face additional stressors related to basic needs, which compounds the pressure of high-stakes testing. The absence of gender-based differences in this study challenges the traditional narrative that female students are more susceptible to exam-related stress (Arbabisarjou et al., 2016). This level of equality may suggest that the academic and sociopolitical environment in the West Bank creates a high anxiety floor that affects all students uniformly. This finding is in contrast to several studies that show significant gender disparities, with female students reporting higher levels of anxiety than their male peers (Pagaria, 2020; Yarkwah et al., 2024; Memon et al., 2023; Jirjees et al., 2024).

### 4.3 Academic, lifestyle and related determinants

Beyond demographic categorization, specific academic and lifestyle factors are significantly associated with student well-being. Among these, perceived workload emerges as the most strongly associated factor, indicating that a lower perception of burden is essential for maintaining psychological stability. Mirawdali et al. (2018) revealed that the overload of materials contributes to increased anxiety levels among students. Moreover, GPA was found to have a negative correlation with anxiety; students who achieved higher GPAs experienced significantly lower levels of anxiety compared to those whose GPAs fell within the 60–69 range. Students with low cumulative grade point averages have statistically significantly higher test anxiety (Jirjees et al., 2024). This is consistent with the findings of Brouwer et al. (2022), who found that high grades are a key motivator that can reduce exam anxiety by providing a sense of mastery and reward.

Our findings indicate a significant positive association between physical morbidity, tobacco use, and levels of exam anxiety. This aligns with research by Jarso et al. (2024), which found that smoking exacerbates anxiety, but contrasts with Jirjees et al. (2024), where smoking was found to be a nonsignificant factor. Furthermore, our study highlights that family support acts as a crucial protective factor, in line with the supportive environment framework proposed by Mauliya et al. (2020). However, as noted by Caamaño-Navarrete et al. (2024) and Hanawi et al. (2020), this potential for success is frequently undermined by poor lifestyle choices, such as unhealthy eating habits, insufficient physical activity, and neglect of mental health care.

Concerning physical activity, students who engage in moderate activity for only 1–2 days per week were significantly associated with higher anxiety levels compared to the most active students who exercise 5 or more days a week. This highlights the intricate regional variations in how lifestyle habits affect student mental health (Jarso et al., 2024; Jirjees et al., 2024). Additionally, these findings align with research by Pascoe et al. (2020), which found a link between physical activity and improved mental health outcomes. Cleveland (2017) discovered no significant correlation between physical activity levels and test anxiety among various student populations. The variations indicate that the effectiveness of exercise as an anxiolytic may vary significantly with the frequency of physical activity and specific academic pressures across different geographical regions, suggesting that tailored exercise interventions may be necessary to address the unique needs of students in various contexts, such as those facing high-stress exams or differing cultural attitudes towards exercise.

While the current model recognizes significant correlates of exam anxiety, accounting for 21.2% of the variance, it highlights the influence of unmeasured factors, including psychological traits such as resilience and coping strategies. Research by Li et al. (2026) highlights that dynamic coping traits are indirectly linked to positive mental health outcomes, such as peace of mind and life meaning. Turner et al. (2017) highlight that low levels of resilience are associated with significantly greater psychological distress. External institutional factors further shape these internal traits. Wadi et al. (2022) defined external institutional factors that further influence these internal psychological characteristics. Their framework categorizes stressors as student (e.g., negative thoughts vs. self-care), academic resources (e.g., heavy curriculum), and examiner (e.g., criticism vs. feedback). Yeo and MengOng (2020) reported higher exam anxiety due to poor time management and preparation. These factors are further closely linked by a macro-level sociopolitical crisis. As Shehada (2025) notes, the ongoing conflict significantly affects the Palestinian education system, leading to widespread anxiety, depression, and post-traumatic stress disorder.

### 4.4 Limitations

This study is subject to several limitations. First, the cross-sectional nature of the research facilitates the identification of correlations but precludes any definitive conclusions regarding causality. Second, there may be selection bias from convenience sampling that limits generalizability, as well as potential social desirability bias from self-report measures. Lastly, the reliance on a single data collection method may not have captured the full complexity of the phenomenon, suggesting a need for future qualitative or longitudinal research. Future research should focus on methodological triangulation. Specifically, it would be beneficial to integrate objective physiological measures, such as cortisol levels or heart rate variability, along with qualitative interviews and survey data. This approach would offer a more comprehensive and validated understanding of the student experience.

### 4.5 Implications

The findings highlight the need for creative, crisis-resistant strategies to provide mental health interventions in conflict zones. To move beyond general screening, universities ought to establish a digital intervention program directly integrated into student portals, including immediate access to mental health resources, counseling options, and peer support networks specifically designed for students in war zones. This program should not only enhance awareness of exam anxiety symptoms but also provide practical coping tools, such as asynchronous cognitive-behavioral modules that students can access during city closures or restricted mobility. University officials should consider reforming academic assessments by implementing continuous assessment models. These models would redistribute grading weights, thereby alleviating the psychological burden associated with high-stakes final exams. Regarding clinical support, we propose a “task-shifting” model in which mental health practitioners train senior students in peer psychological first aid, enabling them to provide immediate assistance when professional staff are unavailable due to political violence. Future research should move beyond identifying general distress and focus on specific risk factors. This includes examining the effects of “anticipatory mobility anxiety” as well as the long-term economic stressors associated with the fear of war.

## 5 Conclusion

The current study confirms that exam anxiety is common among Palestinian university students and significantly correlates with lower academic success. Occurring within a framework of chronic sociopolitical and economic adversity, our findings delineate that academic overload and disciplinary demands are associated with this anxiety and are linked to broader social and psychological stressors. The results highlight the protective role of family support and healthy lifestyle habits, specifically regular physical activity, in mitigating anxiety levels, particularly in the context of the unique challenges faced by Palestinian university students.

## Data Availability

The original contributions presented in the study are included in the article/Supplementary material, further inquiries can be directed to the corresponding author.
