# Supplementary material for: Exam anxiety and associated factors among Palestinian university students
Source: Front Psychol. 2026 May 26;17:1776930. doi: 10.3389/fpsyg.2026.1776930 (PMC13246688; doi:10.3389/fpsyg.2026.1776930)
Supplement: Supplementary file 1 [file Data_Sheet_1.pdf]

Table S1.

Multiple Comparisons of Mean Exam Anxiety Scores across Study Variables using Tukey HSD.

Variables	Categories	N	Subset for alpha = 0.05		
			1	2	3
30 min vigorous physical activity, a [1]	5+ days	374	23.95		
	3-4 days	729	33.73	33.73	
	0 day	78		35.58	35.58
	1-2 days	338			36.82
	Sig.		0.614	0.190	0.543
Overload, a [2]	Not true	532	30.00		
	mostly true	674		35.24	
	very true	313			39.26
	Sig.		1.000	1.000	1.000
Grads point Average, a [3]	80-89	693	32.86		
	90 and higher	344	33.27		
	70-79	353		36.20	
	60-69	129			38.82
	Sig.		0.955	1.000	1.000
Institutional affiliation, a [4]	Hebron University	434	32.72		
	An-Najah University	586		34.74	
	Al-Quds University	499		34.96	
	Sig.		1.000	0.921	
Study field, a [5]	Technical and Applied	194	30.48		
	Humanities and Law	475	31.81		
	Sciences	530		35.19	
	Medicine and Health	320			38.53
	Sig.		0.237	1.000	1.000
Family monthly salary, a [6]	More than 5000 NIS	397	33.16		
	2500-5000 NIS	632	34.22	34.22	
	Less than 2500 NIS	490		35.13	
	Sig.		0.183	0.281	

- a [1] : Uses Harmonic Mean Sample Size = 201.769
- a [2] : Uses Harmonic Mean Sample Size = 457.438
- a [3] : Uses Harmonic Mean Sample Size = 267.831
- a [4] : Uses Harmonic Mean Sample Size = 498.783
- a [5] : Uses Harmonic Mean Sample Size = 325.953
- a [6] : Uses Harmonic Mean Sample Size = 488.441
- b. The group sizes are unequal. The harmonic mean of the group sizes is used. Type I error levels are not guaranteed.

Table S2

General linear regression Analysis and Partial Eta Squared of Factors Associated with Exam Anxiety among Palestinian University Students

Parameter	B	Std. Error	t	Sig.	95% Confidence Interval Lower Bound	95% Confidence Interval Upper Bound	Partial Eta Squared
Intercept	35.877	1.226	29.256	.000	33.472	38.283	.363
[University=1.0]	.172	.566	.304	.761	-.939	1.282	.000
[University=2.0]	1.400	.557	2.514	.012	.307	2.492	.004
[University=3.0]	0[a]	.	.	.	.	.	.
[Income=1.0]	2.022	.584	3.460	.001	.876	3.168	.008
[Income=2.0]	1.825	.546	3.341	.001	.754	2.897	.007
[Income=3.0]	0[a]	.	.	.	.	.	.
[Fieldstudy4groups=1.00]	4.566	.798	5.723	.000	3.001	6.131	.021
[Fieldstudy4groups=2.00]	3.039	.731	4.157	.000	1.605	4.473	.011
[Fieldstudy4groups=3.00]	1.062	.725	1.466	.143	-.359	2.484	.001
[Fieldstudy4groups=4.00]	0[a]	.	.	.	.	.	.
[GPA=1.0]	-3.026	.914	-3.311	.001	-4.818	-1.233	.007
[GPA=2.0]	-3.224	.874	-3.689	.000	-4.938	-1.509	.009
[GPA=3.0]	-.944	.887	-1.064	.287	-2.685	.796	.001
[GPA=4.0]	0[a]	.	.	.	.	.	.
[Morbidity=1]	1.657	.758	2.185	.029	.170	3.144	.003
[Morbidity=2]	0[a]	.	.	.	.	.	.
[Tobacco=1.0]	1.773	.556	3.191	.001	.683	2.863	.007
[Tobacco=2.0]	0[a]	.	.	.	.	.	.
[Family support=1.00]	-1.814	.535	-3.389	.001	-2.863	-.764	.008
[Family support=2.00]	0[a]	.	.	.	.	.	.
[Physical activity=1.00]	.121	1.072	.113	.910	-1.982	2.223	.000
[Physical activity=2.00]	2.458	.649	3.786	.000	1.184	3.731	.009
[Physical activity=3.00]	.532	.551	.965	.335	-.549	1.613	.001
[Physical activity=4.00]	0[a]	.	.	.	.	.	.
[Overload group =1.00]	-6.805	.647	-10.516	.000	-8.075	-5.536	.069
[Overload group =2.00]	-2.493	.616	-4.049	.000	-3.700	-1.285	.011
[Overload group =3.00]	0[a]	.	.	.	.	.	.

Note. a. This parameter is set to zero because it is redundant.

Table S3

Tests of between-subjects effects and model goodness-of-fit for predictors of exam anxiety

Source	Type III Sum of Squares	df	Mean Square	F	Sig.	Partial Eta Squared
Corrected Model	30867.400[a]	18	1714.856	24.522	.000	.227
Intercept	438155.920	1	438155.920	6265.596	.000	.807
University	513.337	2	256.668	3.670	.026	.005
Income	1014.594	2	507.297	7.254	.001	.010
Fieldstudy4groups	3206.148	3	1068.716	15.283	.000	.030
GPA	1737.256	3	579.085	8.281	.000	.016
Morbidity	333.968	1	333.968	4.776	.029	.003
Tobacco used	712.058	1	712.058	10.182	.001	.007
Family support	803.183	1	803.183	11.485	.001	.008
Physical activity	1189.814	3	396.605	5.671	.001	.011
Overload	9024.403	2	4512.201	64.524	.000	.079
Error	104825.735	1499	69.930			
Total	1914108.000	1518				
Corrected Total	135693.136	1517				
a. R Squared = 0.217 (Adjusted R Squared = 0.212)						

Note. Exam anxiety is dependent variable

Table S4

Model Summary and Goodness-of-Fit for the Regression Analysis.

Model	R	R^2	Adjusted R^2	Std.Err	Change Statistics					Durbin-Watson
					R^2 Change	F Change	df1	df2	Sig.	
1	.460[a]	.211	.212	8.42417	.211	44.897	9	1510	.000	2.016

Note. a. Predictors: constant, overload, economic status, type of university, medical morbidity, family support, physical activity, tobacco use, grade point average, and field of study. b. Dependent Variable: Exam anxiety

Table S5

Descriptive Statistics and Normality Diagnostics for Exam Anxiety (N = 1,519)

			Statistic	Std. Error
**Exam anxiety**	**Mean**		34.2383	.24281
	95% Confidence Interval for Mean	Lower Bound	33.7620	
		Upper Bound	34.7146	
	5% Trimmed Mean		34.5113	
	Median		35.0000	
	Variance		89.553	
	Std. Deviation		9.46325	
	Minimum		10.00	
	Maximum		50.00	
	Range		40.00	
	Interquartile Range		13.00	
	Skewness		-.391	.063
	Kurtosis		-.445	.125

Table S6

Residual Statistics for Outlier Diagnostics and Influence Measures

	Minimum	Maximum	Mean	Std. Deviation
Predicted Value	24.4324	48.7188	34.2279	4.34773
Std. Predicted Value	-2.253	3.333	.000	1.000
Standard Error of Predicted Value	.302	1.192	.666	.156
Adjusted Predicted Value	24.4957	48.7007	34.2284	4.35008
Residual	-29.16778	19.99994	.00000	8.39914
Std. Residual	-3.462	2.374	.000	.997
Stud. Residual	-3.476	2.378	.000	1.000
Deleted Residual	-29.38969	20.07250	-.00047	8.45669
Stud. Deleted Residual	-3.488	2.382	.000	1.001
Mahal. Distance	.956	29.360	8.994	4.723
Cook's Distance	.000	.017	.001	.001
Centered Leverage Value	.001	.019	.006	.003
